# Research on the Mechanism of Guizhi to Treat Nephrotic Syndrome Based on Network Pharmacology and Molecular Docking Technology

**DOI:** 10.1155/2021/8141075

**Published:** 2021-11-27

**Authors:** Dan He, Qiang Li, Guangli Du, Jijia Sun, Guofeng Meng, Shaoli Chen

**Affiliations:** ^1^School of Basic Medicine, Shanghai University of Traditional Chinese Medicine, Shanghai 201203, China; ^2^School of Pharmacy, Shanghai University of Traditional Chinese Medicine, Shanghai 201203, China; ^3^Institute of Interdisciplinary Integrative Medicine Research, Shanghai University of Traditional Chinese Medicine, Shanghai 201203, China

## Abstract

**Objective:**

Nephrotic syndrome (NS) is a common glomerular disease caused by a variety of causes and is the second most common kidney disease. Guizhi is the key drug of Wulingsan in the treatment of NS. However, the action mechanism remains unclear. In this study, network pharmacology and molecular docking were used to explore the underlying molecular mechanism of Guizhi in treating NS.

**Methods:**

The active components and targets of Guizhi were screened by the Traditional Chinese Medicine Systems Pharmacology Database and Analysis Platform (TCMSP), Hitpick, SEA, and Swiss Target Prediction database. The targets related to NS were obtained from the DisGeNET, GeneCards, and OMIM database, and the intersected targets were obtained by Venny2.1.0. Then, active component-target network was constructed using Cytoscape software. And the protein-protein interaction (PPI) network was drawn through the String database and Cytoscape software. Next, Gene Ontology (GO) and pathway enrichment analyses of Kyoto Encyclopedia of Genes and Genomes (KEGG) enrichment analyses were performed by DAVID database. And overall network was constructed through Cytoscape. Finally, molecular docking was conducted using Autodock Vina.

**Results:**

According to the screening criteria, a total of 8 active compounds and 317 potential targets of Guizhi were chosen. Through the online database, 2125 NS-related targets were identified, and 93 overlapping targets were obtained. In active component-target network, beta-sitosterol, sitosterol, cinnamaldehyde, and peroxyergosterol were the important active components. In PPI network, VEGFA, MAPK3, SRC, PTGS2, and MAPK8 were the core targets. GO and KEGG analyses showed that the main pathways of Guizhi in treating NS involved VEGF, Toll-like receptor, and MAPK signaling pathway. In molecular docking, the active compounds of Guizhi had good affinity with the core targets.

**Conclusions:**

In this study, we preliminarily predicted the main active components, targets, and signaling pathways of Guizhi to treat NS, which could provide new ideas for further research on the protective mechanism and clinical application of Guizhi against NS.

## 1. Introduction

Nephrotic syndrome (NS) is a clinical syndrome defined as massive proteinuria, hypoalbuminemia, hyperlipidemia, and edema [1]. According to the epidemiological survey, the incidence of NS is about 2-10/100000, and it mostly occurs in male children [2]. NS greatly affects people's health and life quality with poor prognosis and high recurrence rate [3]. At present, the etiopathogenesis of NS is incompletely understood, but relevant reports have shown that it is related to inflammatory response and immune suppression.

According to traditional Chinese medicine (TCM), NS belongs to “edema,” and the pathogenesis of NS lies in the dysfunction of the lung, spleen, and kidney [4]. TCM has been used to treat kidney disease and its complications, for that it can protect the kidney from dysfunction and delay the renal failure [5]. Guizhi is a Chinese herbal medicine that is commonly used to treat edema [6]. Currently, there are many reports on the pharmacological effects of Guizhi, but it is not comprehensive and systematic in the mechanism studies of Guizhi to treat NS.

Network pharmacology integrates diseases and drugs into the biomolecular network, to predict the active components and the action mechanism [7]. As a new idea approach of TCM research, network pharmacology has been widely applied in the research of the complex network relationship between TCM and disease [8–10].

**Figure 1:**
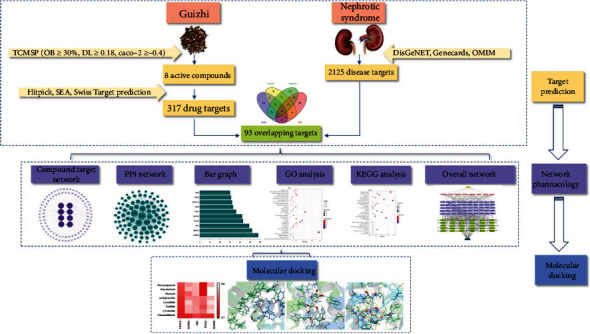
A flow diagram based on a cohesive integration strategy of network pharmacology and molecular docking.

In the present study, network pharmacology and molecular docking were used to explore the potential action mechanism of Guizhi to treat NS. It is hoped to provide theoretical foundation and scientific evidence for the clinical treatment of NS, and the workflow of our study is shown in Figure 1.

## 2. Materials and Methods

### 2.1. Screening of Active Compounds

The active compounds of Guizhi were searched by TCMSP database (https://tcmspw.com/tcmsp.php). The PubChem ID, molecular formula, and canonical SMILES of each component were collected through PubChem database (https://pubchem.ncbi.nlm.nih.gov/). And the main active components were obtained by the screening criteria of oral bioavailability (OB) ≥ 30%, drug − like (DL) ≥ 0.18, and cell permeability (Caco − 2) ≥ −0.4.

### 2.2. Predicting Drug Targets

The targets of active compounds of Guizhi were predicted by Hitpick (http://mips.helmholtz-muenchen.de/hitpick/cgi-bin/index.cgi?content=help.html), SEA (http://sea.bkslab.org/), and Swiss Target Prediction database (http://swisstargetprediction.ch/). The duplicates were deleted after the predicted targets of the 3 databases were merged.

### 2.3. Screening of Disease Targets

Using “Nephrotic syndrome” and “Adriamycin Nephropathy” as the keywords, the NS-related targets were collected from DisGeNET, GeneCards, and OMIM databases. After the removal of repeated targets, Venny2.1.0 was used to screening the intersection of drug targets and disease targets to obtain the potential targets of Guizhi in the treatment of NS.

### 2.4. Construction of Active Component-Target Network

The potential targets were imported into Cytoscape to construct the components-target-network.

### 2.5. Construction of PPI Network

The PPI network was constructed using the String database and Cytoscape software. In this process, the potential targets were input into the String database to obtain the protein interactions, and the interactions were visualized by Cytoscape software in a form of PPI network.

### 2.6. GO and KEGG Pathway Enrichment Analysis

DAVID database was used for GO and KEGG pathway enrichment analysis. The GO and KEGG enriched terms were collected for biological process (BP), cell component (CC), and molecular function (MF), at a cutoff of *P* < 0.05, and the corresponding bubble diagram were drawn.

### 2.7. Construction of Overall Network

The active compounds of Guizhi, the top 30 KEGG signaling pathways, and the corresponding targets were used to construct the drug-compound-target-pathway-disease network through Cytoscape.

### 2.8. Molecular Docking

The top 5 important targets with high network connection degrees were selected for molecular docking analysis using Autodock Vina. The smaller the binding energy (affinity) was, the more stable the interaction between the target protein and the active ingredient was.

## 3. Results

### 3.1. Active Compounds of Guizhi

A total of 220 compounds were collected from the TCMSP database, with OB ≥ 30%, DL ≥ 0.18, and Caco − 2 ≥ −0.4, and 7 active compounds were identified. Cinnamaldehyde, OB = 31.99%, DL = 0.02, and Caco − 2 = 1.35, was not included in the results of TCMSP screening. However, our previous study found that cinnamaldehyde might be an important active compound of Guizhi [11]. Therefore, cinnamaldehyde was selected as a candidate active component in this study, as shown in Table 1.

### 3.2. Drug Target Prediction

8 active components of Guizhi were input into Hitpick, SEA, and Swiss Target Prediction databases. After combining the predicted targets from the 3 databases, the duplicates were deleted, and a total of 317 potential targets were selected.

### 3.3. Disease Target Prediction

**Figure 2:**
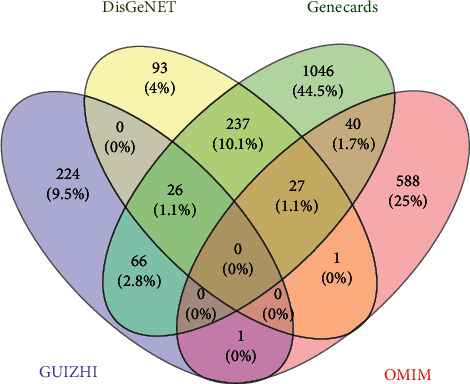
The Venny plot of 93 potential targets.

The keywords “Nephrotic syndrome” and “Adriamycin Nephropathy” were searched in DisGeNET, GeneCards, and OMIM databases. A total of 2125 targets were identified. Venny 2.1.0 was used to intersect disease targets with drug targets. Finally, 93 potential targets (Figure 2) were selected and further confirmed by UniProt database, as shown in Table 2.

### 3.4. Analysis of Active Component-Target Network

**Figure 3:**
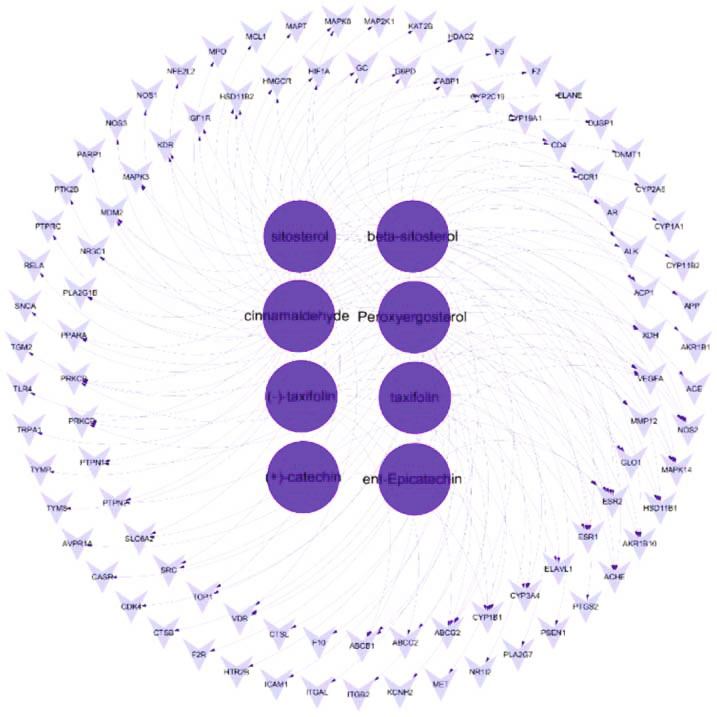
The active component-target network. The network formed with 101 nodes and 169 edges. The dark purple circles represented active compounds; the light purple inverted triangles represented intersecting targets. The edges represented the connection between active component and targets.

The 93 potential targets were analyzed by Cytoscape to construct the active component-target interaction network (Figure 3). The result included 101 nodes and 169 edges. And different components indicated different targets. Among them, the degree values of beta-sitosterol, sitosterol, cinnamaldehyde, and peroxyergosterol were 37, 37, 36, and 29, respectively (Table 3), which might be the important active components in the network.

### 3.5. PPI Network Analysis

**Figure 4:**
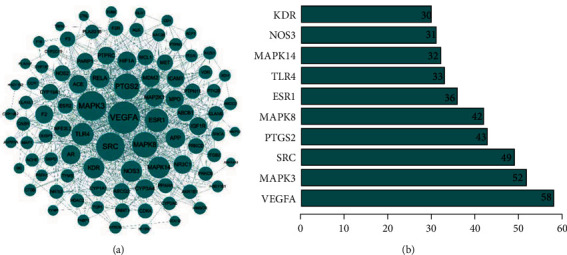
The PPI network diagram (a) and the bar graph of the top 10 intersecting targets with degree values in PPI network (b). In PPI network, nodes represented intersecting targets, and edges represented interactions between targets, and the size reflected the value of degree. In the bar graph, the top 10 targets were selected according to the degree value.

A total of 93 nodes and 1478 edges were involved in the PPI network (Figure 4(a)). The bar chart of the top 10 target proteins was drawn based on the degree value (Figure 4(b)). Among them, VEGFA, MAPK3, SRC, PTGS2, and MAPK8 degree values were 58, 52, 49, 43, and 42, respectively, which were the core nodes of the network, suggesting that Guizhi might play a significance role in the protection of NS through them.

### 3.6. GO and KEGG Analysis

**Figure 5:**
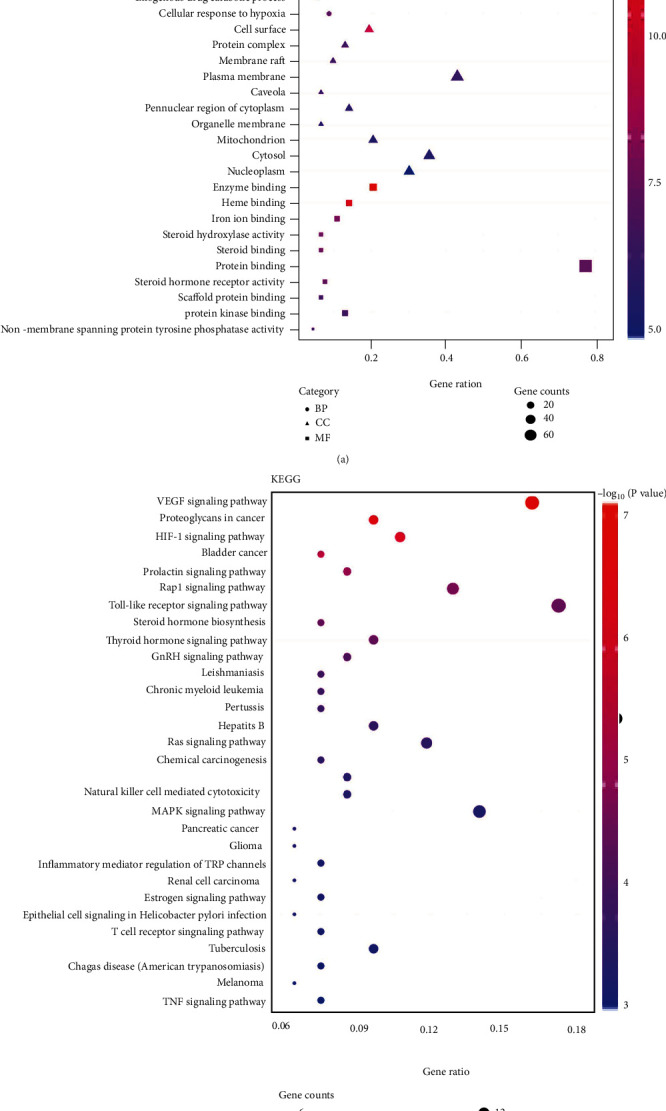
Go (a) and KEGG enrichment analysis (b). The top 10 items of BP, CC, and MF and the top 30 KEGG signal pathways were selected according to the *P* value to draw the GO and KEGG bubble diagram. The color of the bubbles changes from purple to red indicating that the *P* value decreases from large to small. Gene ratio is the number of targets that located in the pathway. The higher the gene ratio is, the more targets were enriched.

93 potential targets were analyzed using DAVID 6.8, and the GO terms (BP, CC, and MF) and KEGG signaling pathway were selected. Targets in the BP were closely related to response to organic substance and positive regulation of molecular function and response to hormone stimulus. In the CC, Guizhi had great effect on cell surface, cell fraction, and plasma membrane part. At the MF level, drug components of Guizhi were mainly related to steroid binding, heme binding, and tetrapyrrole binding (Figure 5(a)). A total of 71 KEGG pathways were mainly involved, including VEGF, Toll-like receptor, and MAPK signaling pathway (Figure 5(b)).

### 3.7. Overall Network Analysis

**Figure 6:**
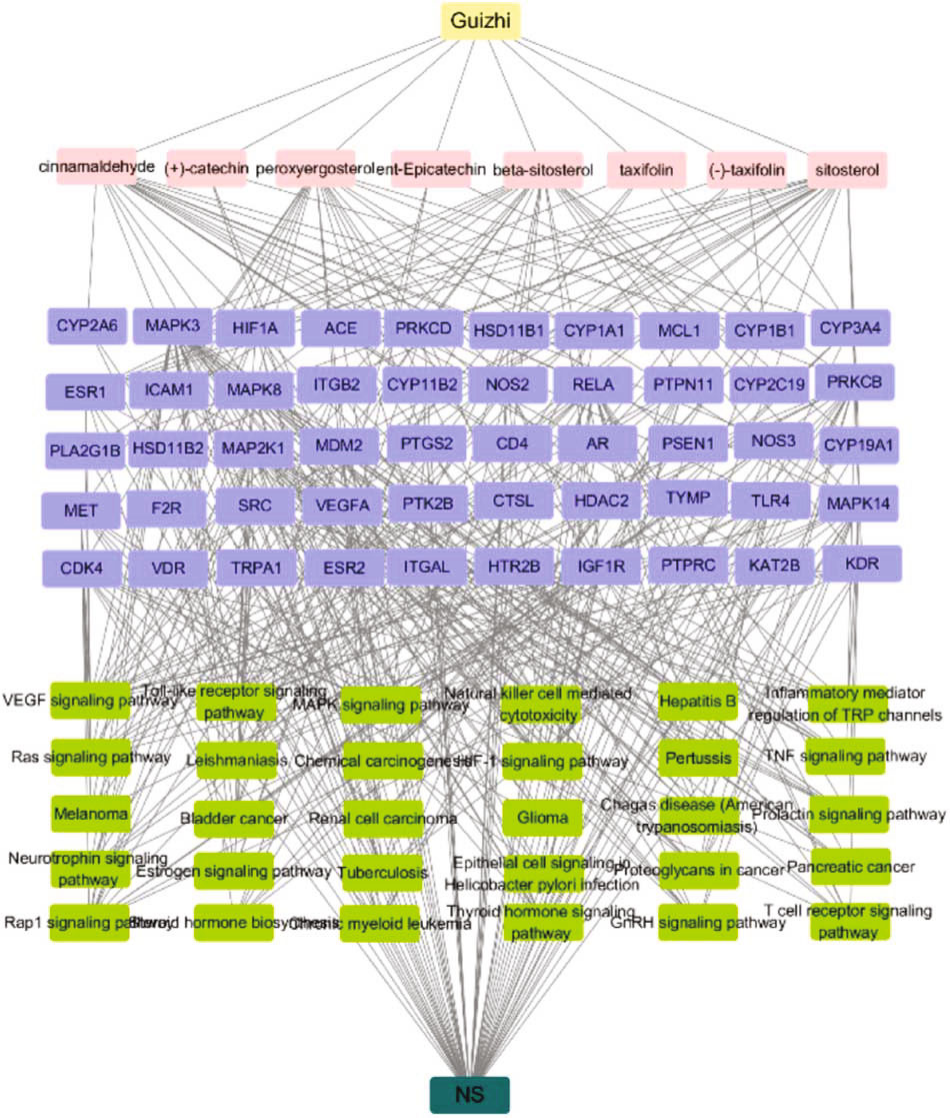
The overall network of the top 30 significant KEGG signaling pathways with their corresponding targets. The yellow represents Guizhi, pink represents active compounds, purple represents core targets, green represents signaling pathways, and dark green represents NS.

To further investigate the molecular mechanism of Guizhi against NS, overall network was constructed based on the top 30 significant KEGG signaling pathways and their corresponding targets (Figure 6). 90 nodes (1 drug, 8 compound, 50 targets, 30 pathways, and 1disease) were contained in this network. Among these targets, MAPK3, MAPK8, MAPK14, VEGFA, and TLR4 were identified as high-degree targets, and in these pathways, Toll-like receptor, VEGF, and MAPK signaling pathways were the most important signaling pathways. Therefore, the network analysis suggests that the action mechanism of Guizhi to treat NS might be related to Toll-like receptor, VEGF, and MAPK signaling pathways.

### 3.8. Molecular Docking

**Figure 7:**
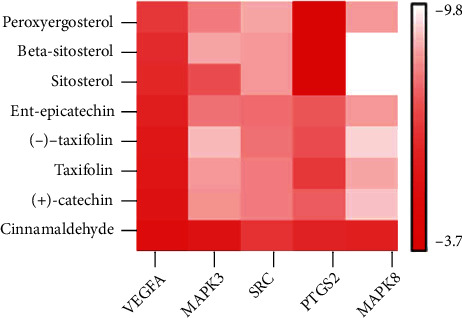
The binding energy of the main active components of Guizhi and core targets.

**Figure 8:**
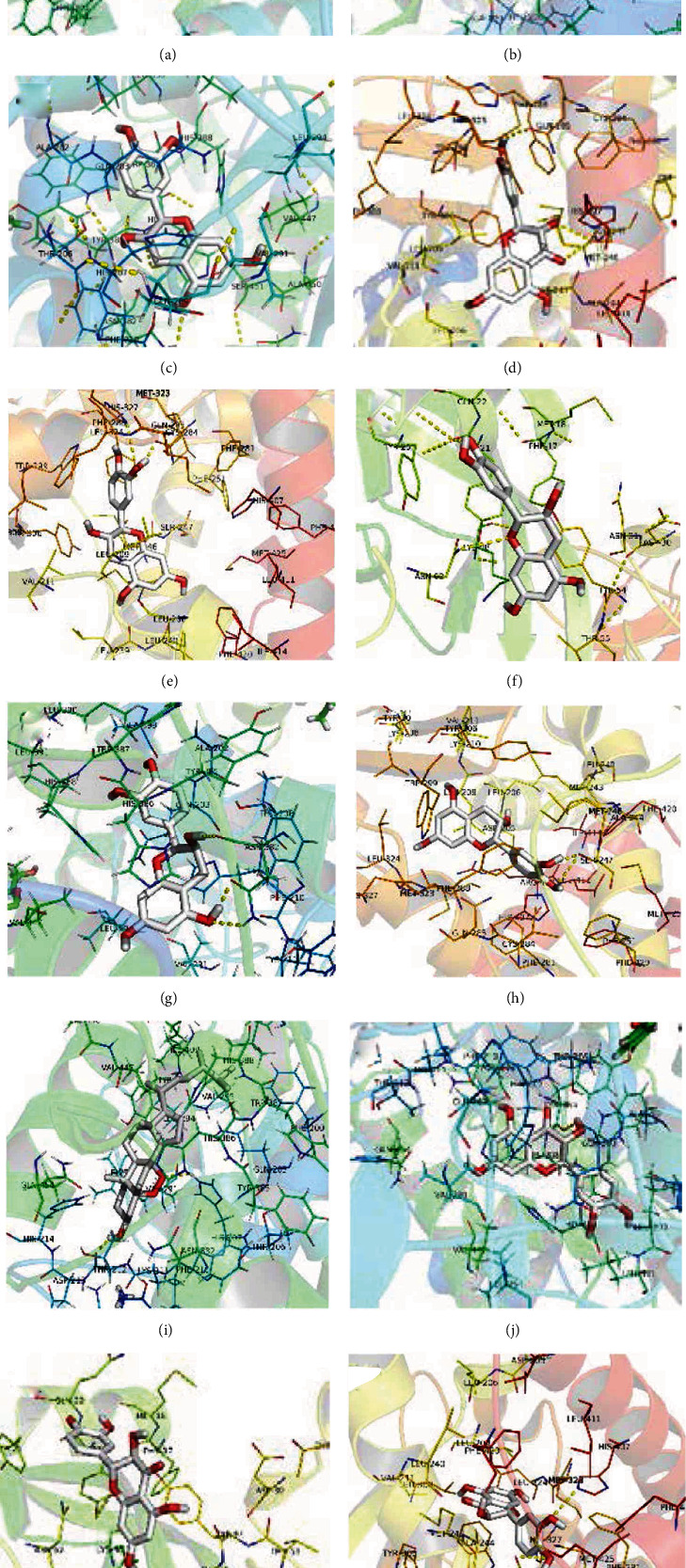
The molecular docking of active compounds and core targets: (a) cinnamaldehyde-PTGS2, (b) (-)-taxifolin-PTGS2, (c) (+)-catechin-PTGS2, (d) (-)-taxifolin-VEGFA, (e) (+)-catechin-SRC, (f) (+)-catechin-VEGFA, (g) ent-Epicatechin-PTGS2, (h) ent-Epicatechin-SRC, (i) peroxyergosterol-PTGS2, (j) taxifolin-PTGS2, (k) taxifolin-VEGFA, and (l) taxifolin-SRC.

The top 5 targets, including VEGFA, MAPK3, SRC, PTGS2, and MAPK8 (Table 4) in the PPI network were analyzed for the molecular docking with the active compounds of Guizhi. It was found that the top 5 targets had good binding affinity with the active components of Guizhi (Table 5; Figure 7). And the binding site of compounds-targets are shown in Figure 8.

## 4. Discussion

NS is a common glomerular disease caused by various etiologies and is the second most common kidney disease after acute glomerulonephritis. At present, Western medicine mainly focuses on glucocorticoids, cytotoxic drugs, and immunosuppressants for NS [1], such as glucocorticoids, cyclophosphamide, and cyclosporine, which can achieve certain efficacy. However, hormone therapy is prone to infection, hormone resistance [12], withdrawal, and relapse [3], which eventually lead to chronic terminal renal failure [13, 14]. Therefore, to explore the pathogenesis of NS and to find safe and effective treatment drugs are urgent problems [15].

Wulingsan is a classic prescription for NS, which has obvious advantages in improving urinary system diseases. Guizhi is an important component of Wulingsan, which has the pharmacological activities of diuresis, improving blood circulation and dilating blood vessels. Our preliminary study found that Guizhi is indeed an important drug of Wulingsan with the protective effect on rats with adriamycin-induced nephropathy [11]. However, the action mechanism remains unknown.

In order to further explore the potential mechanism of Guizhi in treating NS and provide more evidence for clinical treatment, the main active components and targets of Guizhi, as well as the possible signaling pathways of Guizhi to treat NS, were predicted through network pharmacology and molecular docking.

In the active component-target network, the degree values of beta-sitosterol, sitosterol, cinnamaldehyde, and peroxyergosterol were much higher than those of other components, which were 37, 37, 36, and 29, respectively, suggesting that they were the key active components in the treatment of NS.

Among them, beta-sitosterol and sitosterol belong to plant sterols. Studies have found that beta-sitosterol has antihyperlipemia, anti-inflammatory, and immunomodulatory effects and can treat cholesterol, proteinuria, and edema [16]. Cinnamaldehyde is an organic compound of olefine aldehyde, and it is the main component of Guizhi that plays a diuretic role. Pharmacological studies have shown that cinnamaldehyde has a variety of pharmacological activity of anti-inflammatory [17], antitumor [18], hypotensive [19], lipid-lowering [20], hypoglycemic [21], and vascular endothelial protection [22], that is playing a protective role on kidney in various aspects. Our previous study also found that cinnamaldehyde had a protective effect on renal function in adriamycin nephropathy rat [11]. Peroxyergosterol is a kind of relatively rare sterol, with antioxidant [23], antibacterial [24], immunosuppressive [25], antitumor [26], and other activities and can repair damaged kidney cells through antioxidant action. In conclusion, beta-sitosterol, sitosterol, cinnamaldehyde, and peroxyergosterol might be the main pharmacodynamic basis of Guizhi against NS.

In the PPI network, VEGFA, MAPK3, SRC, PTGS2, and MAPK8 had higher degrees than others, indicating that Guizhi might achieve the protective effect on the kidney through the above targets.

VEGFA is a receptor in vascular endothelial cells that can induce endothelial cell differentiation and proliferation [27]. It is an important molecule that maintains the function of the glomerular filtration barrier and plays an important role in renal microangiogenesis. The study has shown that the upregulation of VEGF expression is closely related to the occurrence of proteinuria [28]. Its signal conduction runs through the whole life process of podocytes and glomerular vascular endothelial cells. MAPK3 and MAPK8 are both mitogen-activated protein kinases that can mediate the progress of differentiation, proliferation, and apoptosis [29, 30]. SRC is a nonreceptor protein with tyrosine protein kinase activity and plays an important role in mitosis and proliferation in normal cell [31, 32]. SRC can participate in the process of cell differentiation, proliferation, and apoptosis through the MAPK signaling pathway and plays a crucial role in the inflammation and autoimmune diseases. PTGS2 is significant in inhibiting the development of excessive fibrosis and inflammatory response. When cells are stimulated, the expression of PTGS2 is rapidly upregulated, which catalyzes arachidonic acid to produce a variety of prostaglandins and produces anti-inflammatory and antifibrotic effects, thus achieving the protective effect on the kidney [33]. Therefore, the targets of VEGFA, MAPK3, SRC, PTGS2, and MAPK8 might play an essential role in the protective effect of Guizhi against NS.

In KEGG and overall network analysis, the key targets were mainly involved in VEGF, Toll-like receptor, and MAPK signaling pathway, which were highly correlated with renal disease, suggesting that the action mechanism of Guizhi to treat NS might be related to VEGF, Toll-like receptor, and MAPK signaling pathways.

VEGF signaling pathway plays an irreplaceable role in the whole process of angiogenesis and is directly related to the occurrence of hypertension. When the endothelial function of patients with hypertensive is impaired, blood pressure will significantly increase, and proteinuria is aggravated. Studies have found that the inhibition of VEGF signaling pathway can lead to large amounts of proteinuria accompanied by irreversible renal impairment [34]. In recent years, the role of the innate immune Toll-like receptor (TLR) in kidney disease has attracted more and more attention [35] and is involved in the innate immune and inflammatory response [36]. Studies have shown that TLR can be expressed in a small amount in renal mesangial cells, renal tubular epithelial cells, and podocytes [37]. The increase of TLR expression can cause the pathophysiological changes such as the upregulation of inflammatory factors and cell apoptosis [38]. MAPK signaling pathway is a stress pathway of cellular functional activities, plays a vital role in acute and chronic inflammation, and participates in physiological processes such as cell growth, development, differentiation, and apoptosis [39]. Activation of MAPK signaling pathway can not only trigger renal inflammation [40] but also lead to podocyte injury [41]. Studies have shown that MAPK signaling pathway can cause proteinuria and glomerulosclerosis through oxidative stress, triggering inflammatory factors and activating inflammatory pathways [42]. Munkonda et al. found that MAPK can also promote proximal tubule fibrosis, thus mediating the development of renal disease [43].

Based on the network pharmacology results, molecular docking was performed to verify the interactions between the 8 active components of Guizhi and the key targets. The docking results showed that all the compounds had good binding activities with the targets, indicating that they may play an important role in Guizhi to treat NS.

## 5. Conclusion

In conclusion, the potential mechanism of Guizhi to treat NS was predicted based on the network pharmacology and molecular docking. The results indicated that the underlying mechanism of Guizhi in treating NS may be related to VEGF, Toll-like receptor, and MAPK signaling pathway. However, the transmission of each signal pathway is complicated, and the final mechanism needs to be further verified by subsequent experiments.

## Figures and Tables

**Table 1:** The main active compounds of Guizhi.

Mol ID	Molecule name	OB (%)	DL	Caco-2
MOL000073	Ent-Epicatechin	48.96	0.24	0.02
MOL000358	Beta-sitosterol	36.91	0.75	1.32
MOL000359	Sitosterol	36.91	0.75	1.32
MOL000492	(+)-catechin	54.83	0.24	-0.03
MOL000991	Cinnamaldehyde	31.99	0.02	1.35
MOL001736	(-)-taxifolin	60.51	0.27	-0.24
MOL004576	Taxifolin	57.84	0.27	-0.23
MOL011169	Peroxyergosterol	44.39	0.82	0.86

**Table 2:** 93 potential targets and UniProt information.

No.	Gene names	Protein names	UniProt ID
1	ABCB1	ATP-dependent translocase ABCB1	P08183
2	ABCC2	ATP-binding cassette subfamily C member 2	Q92887
3	ABCG2	Broad substrate specificity ATP-binding cassette transporter ABCG2	Q9UNQ0
4	ACE	Angiotensin-converting enzyme	P12821
5	ACHE	Acetylcholinesterase	P22303
6	ACP1	Low molecular weight phosphotyrosine protein phosphatase	P24666
7	AKR1B1	Aldo-keto reductase family 1 member B1	P15121
8	AKR1B10	Aldo-keto reductase family 1 member B10	O60218
9	ALK	ALK tyrosine kinase receptor	Q9UM73
10	APP	Amyloid-beta precursor protein	P05067
11	AR	Androgen receptor	P10275
12	AVPR1A	Vasopressin V1a receptor	P37288
13	CASR	Extracellular calcium-sensing receptor	P41180
14	CCR1	C-C chemokine receptor type 1	P32246
15	CD4	T-cell surface glycoprotein CD4	P01730
16	CDK4	Cyclin-dependent kinase 4	P11802
17	CTSB	Cathepsin B	P07858
18	CTSL	Procathepsin L	P07711
19	CYP11B2	Cytochrome P450 11B2	P19099
20	CYP19A1	Cytochrome P450 19A1	P11511
21	CYP1A1	Cytochrome P450 1A1	P04798
22	CYP1B1	Cytochrome P450 1B1	Q16678
23	CYP2A6	Cytochrome P450 2A6	P11509
24	CYP2C19	Cytochrome P450 2C19	P33261
25	CYP3A4	Cytochrome P450 3A4	P08684
26	DNMT1	DNA (cytosine-5)-methyltransferase 1	P26358
27	DUSP1	Dual specificity protein phosphatase 1	P28562
28	ELANE	Neutrophil elastase	P08246
29	ELAVL1	ELAV-like protein 1	Q15717
30	ESR1	Estrogen receptor	P03372
31	ESR2	Estrogen receptor beta	Q92731
32	F10	Coagulation factor X	P00742
33	F2	Coagulation factor II	P00734
34	F2R	Coagulation factor II receptor	P25116
35	F3	Coagulation factor III	P13726
36	FABP1	Fatty acid-binding protein 1	P07148
37	G6PD	Glucose-6-phosphate 1-dehydrogenase	P11413
38	GC	Group-specific component	P02774
39	GLO1	Glyoxalase I	Q04760
40	HDAC2	Histone deacetylase 2	Q92769
41	HIF1A	Hypoxia-inducible factor 1-alpha	Q16665
42	HMGCR	3-Hydroxy-3-methylglutaryl-coenzyme A reductase	P04035
43	HSD11B1	Corticosteroid 11-beta-dehydrogenase isozyme 1	P28845
44	HSD11B2	Corticosteroid 11-beta-dehydrogenase isozyme 2	P80365
45	HTR2B	5-Hydroxytryptamine receptor 2B	P41595
46	ICAM1	Intercellular adhesion molecule 1	P05362
47	IGF1R	Insulin-like growth factor 1 receptor	P08069
48	ITGAL	Integrin alpha-L	P20701
49	ITGB2	Integrin beta-2	P05107
50	KAT2B	Histone acetyltransferase KAT2B	Q92831
51	KCNH2	Potassium voltage-gated channel subfamily H member 2	Q12809
52	KDR	Kinase insert domain receptor	P35968
53	MAP2K1	Dual specificity mitogen-activated protein kinase kinase 1	Q02750
54	MAPK14	Mitogen-activated protein kinase 14	Q16539
55	MAPK3	Mitogen-activated protein kinase 3	P27361
56	MAPK8	Mitogen-activated protein kinase 8	P45983
57	MAPT	Microtubule-associated protein tau	P10636
58	MCL1	Induced myeloid leukemia cell differentiation protein Mcl-1	Q07820
59	MDM2	E3 ubiquitin-protein ligase Mdm2	Q00987
60	MET	Hepatocyte growth factor receptor	P08581
61	MMP12	Matrix metalloproteinase-12	P39900
62	MPO	Myeloperoxidase	P05164
63	NFE2L2	Nuclear factor erythroid 2-related factor 2	Q16236
64	NOS1	Peptidyl-cysteine S-nitrosylase NOS1	P29475
65	NOS2	Peptidyl-cysteine S-nitrosylase NOS2	P35228
66	NOS3	NOS type III	P29474
67	NR1I2	Nuclear receptor subfamily 1 group I member 2	O75469
68	NR3C1	Nuclear receptor subfamily 3 group C member 1	P04150
69	PARP1	Poly [ADP-ribose] polymerase 1	P09874
70	PLA2G1B	Phosphatidylcholine 2-acylhydrolase 1B	P04054
71	PLA2G7	Platelet-activating factor acetylhydrolase	Q13093
72	PPARA	Peroxisome proliferator-activated receptor alpha	Q07869
73	PRKCB	Protein kinase C beta type	P05771
74	PRKCD	Protein kinase C delta type	Q05655
75	PSEN1	Presenilin-1	P49768
76	PTGS2	Prostaglandin G/H synthase 2	P35354
77	PTK2B	Protein-tyrosine kinase 2-beta	Q14289
78	PTPN11	Tyrosine-protein phosphatase nonreceptor type 11	Q06124
79	PTPN2	Tyrosine-protein phosphatase nonreceptor type 2	P17706
80	PTPRC	Receptor-type tyrosine-protein phosphatase C	P08575
81	RELA	Transcription factor p65	Q04206
82	SLC6A2	Sodium-dependent noradrenaline transporter	P23975
83	SNCA	Alpha-synuclein	P37840
84	SRC	Protooncogene tyrosine-protein kinase Src	P12931
85	TGM2	Protein-glutamine gamma-glutamyltransferase 2	P21980
86	TLR4	Toll-like receptor 4	O00206
87	TOP1	DNA topoisomerase 1	P11387
88	TRPA1	Transient receptor potential cation channel subfamily A member 1	O75762
89	TYMP	Thymidine phosphorylase	P19971
90	TYMS	Thymidylate synthase	P04818
91	VDR	Vitamin D3 receptor	P11473
92	VEGFA	Vascular endothelial growth factor A	P15692
93	XDH	Xanthine dehydrogenase/oxidase	P47989

**Table 3:** Degree value of 8 main active components of Guizhi.

Mol ID	Molecule name	Degree
MOL000358	Beta-sitosterol	37
MOL000359	Sitosterol	37
MOL000991	Cinnamaldehyde	36
MOL011169	Peroxyergosterol	29
MOL001736	(-)-taxifolin	12
MOL004576	Taxifolin	12
MOL000492	(+)-catechin	3
MOL000073	Ent-Epicatechin	3

**Table 4:** The top 5 targets in PPI network.

No.	Target name	PDB ID	Degree
1	VEGFA	6BFT	58
2	MAPK3	2ZOQ	52
3	SRC	6HTY	49
4	PTGS2	5IKV	43
5	MAPK8	4L7F	42

**Table 5:** Molecular docking results of targets and active components.

Target name	PDB ID	Molecule name	Affinity (kcal/Mol)
VEGFA	6BFT	Peroxyergosterol	-6.8
		Beta-sitosterol	-6.4
		Sitosterol	-6.3
		Ent-Epicatechin	-5.9
		(-)-taxifolin	-5.7
		Taxifolin	-5.6
		(+)-catechin	-5.4
		Cinnamaldehyde	-4.9

MAPK3	2ZOQ	(-)-taxifolin	-9.0
		Beta-sitosterol	-8.7
		Taxifolin	-8.5
		(+)-catechin	-8.4
		Peroxyergosterol	-8.1
		Ent-Epicatechin	-7.9
		Sitosterol	-7.3
		Cinnamaldehyde	-5.4

SRC	6HTY	Peroxyergosterol	-8.7
		Beta-sitosterol	-8.5
		Sitosterol	-8.4
		(+)-catechin	-8.1
		Taxifolin	-8.1
		(-)-taxifolin	-7.9
		Ent-Epicatechin	-7.8
		Cinnamaldehyde	-6.6

PTGS2	5IKV	(+)-catechin	-7.6
		Ent-Epicatechin	-7.5
		(-)-taxifolin	-7.3
		Taxifolin	-6.8
		Cinnamaldehyde	-6.1
		Peroxyergosterol	-3.8
		Beta-sitosterol	-3.7
		Sitosterol	-3.7

MAPK8	4L7F	Beta-sitosterol	-9.8
		Sitosterol	-9.8
		(-)-taxifolin	-9.3
		(+)-catechin	-9.1
		Taxifolin	-8.7
		Ent-Epicatechin	-8.5
		Peroxyergosterol	-8.5
		Cinnamaldehyde	-6.0

## Data Availability

The data used to support the findings of this study are included within the article.
